# Use of generic and essential medicines for prevention and treatment of cardiovascular diseases in Portugal

**DOI:** 10.1186/s12913-017-2401-2

**Published:** 2017-06-29

**Authors:** Helena Gama, Carla Torre, José Pedro Guerreiro, Ana Azevedo, Suzete Costa, Nuno Lunet

**Affiliations:** 1 EPIUnit – Institute of Public Health, University of Porto (ISPUP), Porto, Portugal; 2 Centre for Health Evaluation & Research (CEFAR), National Association of Pharmacies Group, Lisbon, Portugal; 3 Department of Clinical Epidemiology, Predictive Medicine and Public Health, University of Porto Medical School, Porto, Portugal; 4 Departamento de Epidemiologia Clínica, Medicina Preditiva e Saúde Pública, Faculdade de Medicina da Universidade do Porto, Alameda Prof. Hernâni Monteiro, 4200-319 Porto, Portugal

**Keywords:** Cardiovascular system, Pharmacoepidemiology, Generic Drugs, Essential medicines, Portugal

## Abstract

**Background:**

The successful control of cardiovascular diseases at the lowest possible cost requires the use of the most effective and affordable medicines. We aimed to describe the trends in the ambulatory use of medicines for prevention and treatment of cardiovascular diseases [Anatomic Therapeutic Chemical classification system (ATC): C and B01A] in Portugal, between 2004 and 2012, and to estimate the potential for expenditure reduction through changes in patterns of use.

**Methods:**

We analysed sell-out data, expressed as defined daily doses (DDD) and pharmacy retail price (€), from a nationwide database. We estimated potential reduction in expenditures through the increase, up to 90% of the volume of DDD, in the use of generic and essential medicines; the latter were defined according to guidelines from Portugal and another European country.

**Results:**

Overall consumption increased by approximately 50% from 2004 to 2012, reaching nearly 2400 million DDD, whereas expenditure decreased to 753 million € (−31.3% since 2006). Use of generics and essential medicines increased, representing 43.6 and 39.9% of DDD consumption in 2012, respectively. The 40 most used groups of medicines in 2012 accounted for just over 80% of overall consumption; among these, increase in use of generics and essential medicines would have contributed to a saving of 275 million €.

**Conclusions:**

Changes in patterns of consumption of medicines towards a more frequent use of generics, a preferential use of essential medicines and a more rational use of fixed-dose combinations may contribute to a more efficient use of health resources.

## Background

Cardiovascular diseases encompass a wide range of clinical situations affecting the circulatory system in different anatomic locations and are the primary cause of mortality worldwide [1–3]. They account for over 30% of all deaths, mostly due to ischemic heart disease and stroke, and approximately 10% of the overall number of Disability Adjusted Life Years (DALYs). In high income countries the mortality burden of cardiovascular diseases has been declining for decades, reflecting trends towards a better control of cholesterol levels and hypertension at a population level [4–6], as well as more frequent use of evidence-based interventional and pharmacological therapies for acute management and secondary prevention after acute coronary syndromes and stroke [4–8].

In Portugal, the most recent trends in mortality due to cardiovascular diseases parallel declines observed in Western Europe, North America, Japan and other areas of the developed world [3, 9, 10], also reflecting improvements in the awareness and treatment of these conditions [11]. The number of defined daily doses (DDD) of medicines for the cardiovascular system consumed in Portugal increased nearly two-fold between 2000 and 2010, representing just over one-quarter of the overall expenditure on medicines in recent years [12]. Nevertheless, there is a large margin for a more rational use of these medicines, towards a more effective prevention and control of cardiovascular diseases at the lowest possible cost [13–17].

Use of off-patent-generic medicines has contributed to reduce health expenditures throughout Europe [18, 19]. In Portugal, the use of generics has been promoted through several measures, including public information campaigns, price reduction, prescription by international non-proprietary name or promoting generic substitution by pharmacists [20, 21]; in the first month of 2013, generic medicines accounted for nearly 40% of the total volume of sales in the National Health Service [13]. Also, lists of essential medicines have been produced by the World Health Organization (WHO) [22, 23]. Similar instruments are available in more than a hundred countries worldwide, contributing to promote appropriate standards of treatment, to ensure the availability of the most effective medicines and to develop strategies for standard reimbursements [24–26]. No list of essential medicines is available in Portugal. However, the Memorandum of Understanding (MoU) with the International Monetary Fund, the European Commission and the European Central Bank in 2011, signed during the recent financial crisis, requires the publication of clinical guidelines for a more rational use of medicines in different clinical situations [27].

We aimed to describe the trends in the ambulatory use of medicines for prevention and treatment of cardiovascular diseases in Portugal, between 2004 and 2012, and to estimate the potential for cost reduction by increasing use of generics and essential medicines.

## Methods

For the period between 2004 and 2012, we analyzed national data on the medicines dispensed at the community level, obtained from the Pharmacy Sales Information System of the Centre for Health Evaluation & Research (CEFAR) of the Portuguese National Association of Pharmacies. This is a nationwide database, providing representative country and regional estimates of drug dispensing data for all ambulatory care, based on sell-out sample data from 80% of the universe of pharmacies. Drug dispensing data was estimated at the national and regional levels using appropriate weight factors (stratified sampling methodology) according to geographical location and pharmacy income. This database is linked to other administrative databases, gathering information about the characteristics of the medicines dispensed (e.g. generic or non-generic), as well as grouped-population characteristics and regional codes for spatio-temporal analysis.

For the present analyses, we extracted data for each medicine included in the anatomical main group C (Cardiovascular System) or the pharmacological subgroup B01A (Antithrombotic Agents), of the Anatomic Therapeutic Chemical (ATC) classification system [28], distinguishing between generic and non-generic products. Data were expressed as total costs in euro, based on the pharmacy retail price of each medicine, as well as in number of DDD, per year. The ATC/DDD system developed by the World Health Organization (WHO) Collaborating Centre for Drug Statistics Methodology was used to define the DDD, which correspond to the assumed average daily maintenance dose for its main indication use in adults. For substances not included in the ATC/DDD system, as well as for fixed-dose combinations, the daily dose recommended by the marketing authorization holder was used as the DDD.

Since no list of essential medicines is available in Portugal, we selected guidelines from a high income European country as reference, and classified each medicine as essential when it was referred in the “wise list” for essential drug recommendations in ambulatory care issued by the *Stockholm County Pharmaceutical Committee* [29, 30]. We used lists published between 2004 and 2012 as reference to classify the medicines dispensed in each of these years.

We analyzed trends according to the classification of the medicines as generic or non-generic, essential or non-essential, and specifically for 3-hydroxy-3-methylglutaryl coenzyme A reductase inhibitors (statins), as well as for angiotensin-converting enzyme (ACE) inhibitors and angiotensin receptor blockers (ARB).

For the year 2012, we described the 30 groups of medicines with highest consumption in terms of DDD and the 30 ranked higher for expenditure, specifying the contribution of generic and non-generic products to the overall consumption, as well as average cost per DDD for each group.

We estimated potential reduction in expenditures through an increase in the proportion of generics and essential medicines, up to 90%. The simulated scenarios included: 1) replacement of non-generics by generics; 2) replacement of non-essential by essential medicines of the same ATC chemical subgroup; 3) replacement of fixed-dose combinations by associations of two essential medicines with a single active ingredient, recommended for similar clinical situations by the clinical guidelines issued by the Portuguese Directorate-General of Health [27]. We calculated the difference between the observed expenditure in 2012 and the expected expenditure in the simulated scenarios. The latter were obtained by adding the expenditure with medicines not replaced to the product of the number of DDD to be replaced by the mean cost per DDD of the generic substitutes; the mean cost of generic medicines was used as reference whenever these were available. For example, for a medicine whose total sales in 2012 were 100,000 DDD, from which 30% corresponded to generics, with a mean cost of 0.5€ per DDD, and 70% to non-generics, with a mean cost of 1.0 € per DDD, the total expenditure would be (100,000 DDD*0.3*0.5 €) + (100,000 DDD*0.7*1.0 €) = 85,000 €. The replacement of non-generics up to a share of 90% for generics would result in a total expenditure of (100,000 DDD*0.9*0.5 €) + (100,000 DDD*0.1*1.0 €) = 55,000 €, corresponding to the saving of 85,000 € - 55,000 € = 30,000 €. In the simulated scenario in which the replacement was an essential medicine with mean cost of 0.3 per DDD, the total expenditure would be (85,000 €*0.1) + (100,000 DDD*0.9*0.3 €) = 35,500 €, and the estimated cost saving would be 85,000 € - 35,500 € = 49,500 €, corresponding to 58.2% of the observed expenditure in 2012 (49,500 € / 85,000 € * 100).

Data were analysed with STATA, version 11.1.

## Results

**Fig. 1:**
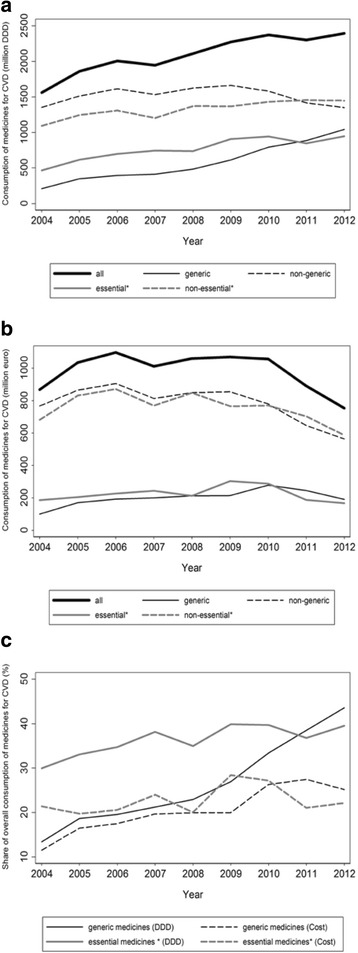
**a**-**c** Trends in the ambulatory use of medicines for the prevention and treatment of cardiovascular diseases between 2004 and 2012, in Portugal. Medicines for CVD – medicines for the prevention and treatment of cardiovascular diseases [Anatomic Therapeutic Chemical classification system (ATC): C and B01A]; DDD – Defined Daily Doses; * essential medicines, according to the “wise list” for essential drug recommendations in ambulatory care issued by the *Stockholm County Pharmaceutical Committee* [45, 46]

Trends in ambulatory use of medicines for the prevention and treatment of cardiovascular diseases between 2004 and 2012 are depicted in Fig. 1. The overall consumption increased by approximately 50%, from just over 1500 million to nearly 2400 million DDD (Fig. 1a), whereas the corresponding cost decreased in the same period. The highest expenditure was observed in 2006 (1096 million €) and the lowest in 2012 (753 million €) (Fig. 1b).

The overall number of DDD from generic medicines increased five-fold in this period, from 210 million in 2004 to 1043 million in 2012, whereas DDD from non-generic medicines fell nearly 20% since 2009 (Fig. 1a). Overall expenditure with generic medicines increased from 100 million € in 2004 to 278 million € in 2010, and was 189 million € in 2012. The highest expenditure with non-generic medicines was in 2006 (904 million €), and decreased to 563 million € in 2012 (Fig. 1b). This resulted in a gradual increase in the proportion of DDD corresponding to generic medicines from 13.4% in 2004 to 43.6% in 2012. The proportion of the overall expenditure, in euro, that was due to generic medicines increased up to 2011 (27.4%) and decreased to 25.2% in 2012 (Fig. 1c).

Regarding use of essential medicines, there was also an increase in the number of DDD from 2004 to 2012 (468 and 947 million DDD, respectively) though less steep than for generic medicines (Fig. 1a). Expenditure with essential medicines was highest in 2009 (303 million €) and declined to 167 million € in 2012 (Fig. 1b). These trends translate into a gradual increase in the proportion DDD of essential medicines up to 2009, from 29.9 to 39.9%, and relatively stable thereafter. The share of overall expenditure, in euro, that was due to essential medicines was highest in 2009, at 28.4%, and decreased to 22.1% in 2012 (Fig. 1c).

**Fig. 2:**
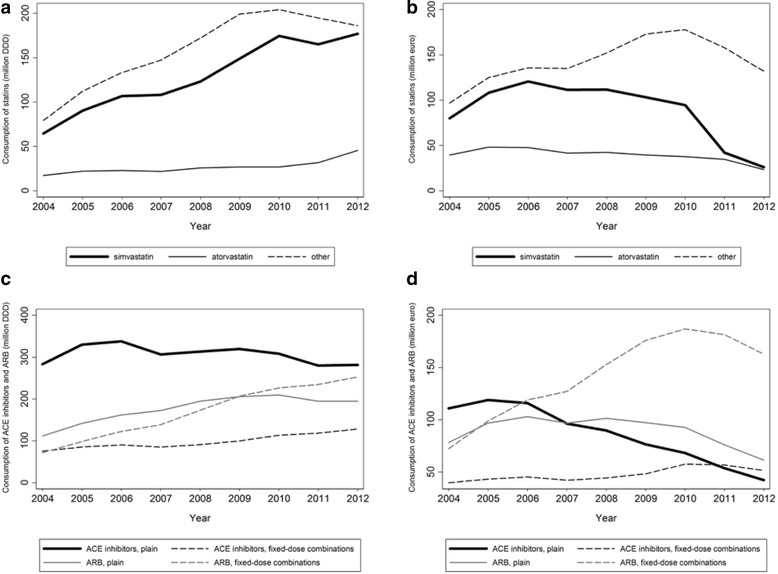
**a**-**d** Trends in the ambulatory use of simvastatin, atorvastatin and other statins (including fixed-dose combinations including statins), as well as of angiotensin converting enzyme (ACE) inhibitors and angiotensin II antagonists (ARB), between 2004 and 2012, in Portugal. ACE inhibitors – Angiotensin-converting enzyme inhibitors [Anatomic Therapeutic Chemical classification system (ATC): C09A and C09B]; ARB – Angiotensin receptor blockers [ATC: C09C and C09D]; DDD – Defined Daily Doses

Figure 2 depicts the trends in the use of statins, ACE inhibitors and ARB, between 2004 and 2012. For statins, the number of DDD consumed increased more than two-fold both for simvastatin (from 64.5 million in 2004 to 176.8 million in 2012) and atorvastatin (from 17.3 million in 2004 to 45.4 million in 2012). Considering remaining medicines from the same chemical subgroup together, consumption increased from 79.3 million DDD in 2004 to 204.3 million DDD in 2010, and decreased to 186.2 million DDD in 2012 (Fig. 2 a). In 2012, expenditures with simvastatin and atorvastatin were 26.0 and 23.4 million €, respectively, and approximately five-fold higher (132.0 million €) for the remaining statins/fixed-dose combinations with statins together (Fig. 2b). Use of plain ACE inhibitors was highest in 2006 (338.1 million DDD) and decreased to just over 280 million DDD in 2012 (Fig. 2c), whereas expenditure with these medicines decreased steeply since 2005, from 119.0 million €, to 42.3 million € in 2012 (Fig. 2d). Consumption of plain ARB was highest in 2010 (209.6 million DDD), and decreased to 194.8 million DDD in 2012 (Fig. 2c), corresponding to approximately 70% of the consumption of ACE inhibitors. Expenditure with plain ARB decreased since 2006, to 61.4 million € in 2012, which is approximately 50% higher than that observed for plain ACE inhibitors (Fig. 2d).

Use of fixed-dose combinations was just over 70 million DDD in 2004 for ACE inhibitors and ARB, and increased up to 2012, to 128.3 million DDD of ACE inhibitors and 252.6 million DDD for ARB (Fig. 2c). Expenditure with these medicines increased up to 2010 and decreased thereafter; in 2012 the volume of sales was still 51.6 million € and 163.0 million € for the fixed-dose combinations of ACE inhibitors and ARB, respectively, which is more than 20 and 165% higher, respectively, than the observed for plain medicines from these groups (Fig. 2d).

**Fig. 3:**
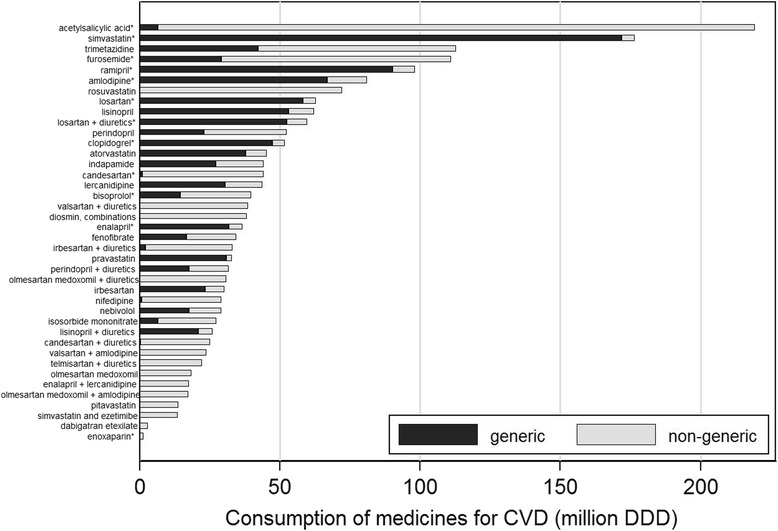
Most consumed medicines (40 subgroups responsible for the highest consumptions, including the top 30 in DDD and the top 30 in value) for the prevention and treatment of cardiovascular diseases in 2012, in Portugal. Medicines for CVD – medicines for prevention and treatment of cardiovascular diseases [Anatomic Therapeutic Chemical classification system (ATC): C and B01A]; DDD – Defined Daily Doses; * essential medicines, according to the “wise list” for essential drug recommendations in ambulatory care issued by the *Stockholm County Pharmaceutical Committee* [45, 46]

**Fig. 4:**
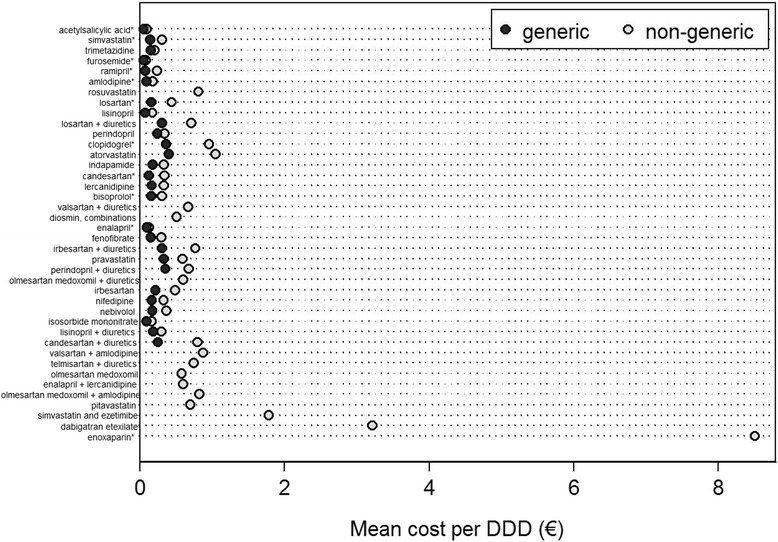
Cost of the most consumed medicines (40 subgroups responsible for the highest consumptions, including the top 30 in DDD and the top 30 in value) generic and non-generic medicines for the prevention and treatment of cardiovascular diseases in 2012, in Portugal. DDD – Defined Daily Doses; * essential medicines, according to the “wise list” for essential drug recommendations in ambulatory care issued by the *Stockholm County Pharmaceutical Committee* [45, 46]

In 2012, the 30 groups of medicines with highest consumption in terms of DDD and the 30 with highest consumption regarding expenditure corresponded to a total of 40 ATC subgroups (Figs. 3 and 4), which accounted for 81.6% of the overall number of DDD and 80.4% of total expenditure. A total of 19 of these medicines corresponded to agents acting on the renin-angiotensin system (ATC: C09), from which 12 were fixed-dose combinations, seven were lipid modifying agents (ATC: C10), four were antithrombotic agents (ATC: B01A) and three were calcium channel blockers (ATC: C08). The remaining were diuretics (ATC: C03), beta blocking agents (ATC: C07), drugs for cardiac therapy (ATC: C01) or vasoprotectives (ATC: C05). Generics were available for 28 of the top 40 medicines, and 12 were classified as essential medicines.

In 2012, the proportion of DDD coming from generics varied widely, from less than 10% for candesartan, nifedipine, acetylsalicylic acid and irbesartan + diuretics to more than 90% for simvastatin, pravastatin, losartan, clopidogrel and ramipril (Fig. 3). The non-generic medicines had a higher mean cost per DDD than the corresponding generics; the relative differences (non-generic in relation to generic medicines) ranged between 32% for enalapril and 301% for ramipril, and absolute differences ranged between 0.025 € for furosemide to 0.642 € for atorvastatin (Fig. 4).

Table 1 presents an estimation of potential for reduction in expenditures through an increase in use of generic and essential medicines. The wide variation in the proportion of generic medicines used and in the cost of generic and non-generic medicines results in a different ranking position when considering the number of DDD or the cost. Among the medicines that rank higher in volume of sales (DDD) than in expenditure, the most pronounced differences were observed for furosemide, ramipril, lisinopril, acetylsalicylic acid, enalapril, amlodipine and simvastatin; for these medicines the consumption expressed in percentage of overall expenditure was less than half the consumption expressed in percentage of the number of DDD. A higher ranking in expenditure, corresponding to a percentage of overall expenditure at least twice higher than the percentage of all DDD consumed, was observed mostly for ACE inhibitors or ARB combined with diuretics or calcium channel blockers (ATC: C09BA, C09DA, C09DB), statins (ATC: C10AA, C10B) and antithrombotic agents (ATC: B01AB, B01AE). A few medicines were responsible for some of the highest expenditures despite ranking below the top 30 in number of DDD, namely the following fixed-dose combinations: simvastatin + ezetimibe (13.7 million DDD, 24.4 million €); valsartan + amlodipine (23.8 million DDD, 20.7 million €); candesartan + diuretics (25.0 million DDD, 19.8 million €); telmisartan + diuretics (22.4 million DDD, 16.8 million €).

**Table 1:** Estimation of the potential for reduction in costs with medicines for prevention and treatment of cardiovascular diseases in Portugal, through an increase in the use of generic medicines and essential medicines among the most consumed (40 subgroups responsible for the highest consumptions, including the top 30 in DDD and the top 30 in value, in 2012)

					Potential expenditure reduction through replacement (up to 90% of the number of DDD)^b^:		
	Overall consumption in 2012				Non-generics by generics	Non-essential by essential	Fixed dose combinations by associations of essential medicines with a single active ingredient
			% from generics				
	Million DDD^l^	Million €	DDD	€	Million € (%)	Million € (%)	Million € (%)
Acetylsalicylic acid^a^	219.1	21.2	2.9	1.7	7.8 (37.0)	---	---
Simvastatin^a^	176.8	26.0	97.4	94.6	---	---	---
Trimetazidine	112.8	20.4	37.3	30.9	2.9 (14.3)	---	---
Furosemide^a^	110.9	8.2	26.3	19.6	1.8 (21.9)	---	---
Ramipril^a^	98.2	8.9	91.8	78.8	---	---	---
Amlodipine^a^	81.0	8.3	82.7	69.8	0.5 (6.6)	---	---
Rosuvastatin	72.1	58.2	0.0	0.0	---	41.5 (71.4)^c^	---
Losartan^a^	62.8	11.1	92.7	81.8	---	---	---
Lisinopril	62.2	5.6	85.4	72.2	0.3 (4.9)	0.7 (13.0)^d^	---
Losartan + diuretics^a^	59.8	21.4	87.7	75.4	0.6 (2.6)	---	8.7 (40.7)^h^
Perindopril	52.5	15.8	43.4	35.5	2.4 (15.0)	10.6 (66.9)^d^	---
Clopidogrel^a^	51.7	21.2	91.9	81.1	---	---	---
Atorvastatin	45.4	23.4	82.9	65.2	2.1 (8.9)	14.3 (60.9)^c^	---
Indapamide	44.2	10.7	61.6	46.8	1.9 (17.8)	---	---
Candesartan^a^	44.1	15.0	2.1	0.8	8.6 (57.7)	---	---
Lercanidipine	43.9	9.6	69.6	53.7	1.5 (15.3)	5.2 (54.2)^e^	---
Bisoprolol^a^	39.8	10.1	36.2	22.8	3.1 (31.1)	---	---
Valsartan + diuretics	38.8	26.0	0.0	0.0	---	---	17.8 (68.4)^h^
Diosmin, combinations	38.3	19.5	0.0	0.0	---	---	---
Enalapril^a^	36.8	3.6	86.1	82.4	0.0 (1.2)	---	---
Fenofibrate	34.6	7.9	48.4	31.9	2.2 (27.4)	---	---
Irbesartan + diuretics	33.1	24.5	6.0	2.5	12.8 (52.0)	---	17.3 (70.5)^h^
Pravastatin	32.9	11.5	94.3	90.3	---	5.4 (47.1)^c^	---
Perindopril + diuretics	31.7	15.9	55.1	39.5	3.5 (22.0)	---	10.9 (68.9)^i^
Olmesartan medoxomil + diuretics	31.0	18.6	0.0	0.0	---	---	12.2 (65.9)^h^
Irbesartan	30.3	8.3	77.8	60.6	1.0 (12.1)	4.2 (50.3)^f^	---
Nifedipine	29.2	9.4	2.1	1.1	4.2 (44.0)	6.2 (65.8)^e^	---
Nebivolol	29.0	7.3	60.5	42.1	1.7 (22.7)	2.4 (32.8)^g^	---
Isosorbide mononitrate	27.5	4.0	23.4	14.6	1.3 (32.7)	---	---
Lisinopril + diuretics	26.0	5.5	80.4	72.2	0.3 (5.0)	---	2.2 (39.7)^i^
Candesartan + diuretics	25.0	19.8	1.1	0.4	12.1 (61.3)	---	14.2 (71.7)^h^
Valsartan + amlodipine	23.8	20.7	0.0	0.0	---	---	14.2 (68.5)^j^
Telmisartan + diuretics	22.4	16.8	0.0	0.0	---	---	11.9 (70.7)^h^
Olmesartan medoxomil	18.4	10.7	0.0	0.0	---	7.6 (71.2)^f^	---
Enalapril + lercanidipine	17.6	10.5	0.0	0.0	---	---	6.8 (65.1)^k^
Olmesartan medoxomil + amlodipine	17.3	14.2	0.0	0.0	---	---	9.5 (67.1)^j^
Pitavastatin	13.9	9.7	0.0	0.0	---	6.7 (68.6)^c^	---
Simvastatin + ezetimibe	13.7	24.4	0.0	0.0	---	---	2.4 (9.7)
Dabigatran etexilate	3.0	9.7	0.0	0.0	---	---	---
Enoxaparin	1.4	11.9	0.0	0.0	---	---	---

*DDD* defined daily doses, *NA* not applicable

^a^essential medicines, according to the “wise list” for essential drug recommendations in ambulatory care issued by the *Stockholm County Pharmaceutical Committee* [45, 46]

^b^We computed the difference between the observed expenditure in 2012 and the expected expenditure in the simulated scenarios. The latter were obtained by adding the expenditure with medicines not replaced to the product of the number of DDD to be replaced by the mean cost per DDD of the generic substitutes. For example, for a medicine whose total sales in 2012 were 100,000 DDD, from which 30% corresponded to generics, with a mean cost of 0.5€ per DDD, and 70% to non-generics, with a mean cost of 1.0 € per DDD, the total expenditure would be (100,000 DDD*0.3*0.5 €) + (100,000 DDD*0.7*1.0 €) = 85,000 €. The replacement of non-generics up to a share of 90% for generics would result in a total expenditure of (100,000 DDD*0.9*0.5 €) + (100,000 DDD*0.1*1.0 €) = 55,000 €, corresponding to the saving of 85,000 € - 55,000 € = 30,000 €. In the simulated scenario in which the replacement was an essential medicine with mean cost of 0.3 per DDD, the total expenditure would be (85,000 €*0.1) + (100,000 DDD*0.9*0.3 €) = 35,500 €, and the estimated cost saving would be 85,000 € - 35,500 € = 49,500 €, corresponding to 58.2% of the observed expenditure in 2012 (49,500 € / 85,000 € * 100)

^c^replacement by simvastatin (a daily dose of 40 mg was considered for half of the DDD replaced)

^d^replacement by ramipril

^e^replacement by amlodipine

^f^replacement by candesartan

^g^replacement by bisoprolol

^h^replacement by losartan and chlortalidone

^i^replacement by ramipril and chlortalidone

^j^replacement by losartan and amlodipine

^k^replacement by ramipril and amlodipine

^l^DDD can be converted in DDD/1000/inhabitants/day by dividing the absolute number of DDD by 3,838,347.774

Under a scenario in which generics are responsible for 90% of the overall consumption an expenditure reduction of 85.2 million € (11.1%) would have been achieved in 2012, mostly when generics account for a small proportion of the overall consumption and/or the differences in the price of generics and non-generics are large. Considering only the top 40 in DDD or cost, expenditure reduction would have been 72.6 million € (9.5%). An additional reduction of 91.8 million € could have been achieved through the option for generic essential medicines of the same ATC chemical subgroups as a replacement for only 11 of the top 40 medicines. The replacement of 12 fixed-dose combinations by associations of two essential medicines with a single active ingredient would have further reduced the expenditure in 98.8 million €. Taken together, these changes in patterns of use of medicines could have contributed to a saving of 275.8 million €, which corresponds to more than one third of expenditure with medicines for prevention and treatment of cardiovascular diseases.

## Discussion

Sales of medicines for ambulatory prevention and treatment of cardiovascular diseases have been increasing in Portugal, along with a higher proportion of generic and essential medicines, and a reduction of overall expenditure.

Steep increases in sales of generic medicines and decreases in non-generics reflects the promotion of low-cost off-patent drug utilization in this country, especially in recent years, principally through recommending prescription by international non-proprietary name [31], which became mandatory in 2012 [32], or promoting generic substitution by pharmacists, in line with the European strategy to reduce pharmaceutical expenditures [18, 33, 34]. This is in accordance with the fact that Portugal had the second lowest generic medicines market share volume among 17 European countries in 2007, close to 10%, reaching approximately 25% during 2012 [13, 34] and to 30% in 2014. [35] Specifically for cardiovascular system drugs, in 2011 generic medicines accounted for just over 40% of those prescribed for ambulatory use through the National Health Service in most Portuguese regions [12], in accordance with our results. Nevertheless, the present study shows that among medicines sold for the ambulatory prevention and treatment of cardiovascular diseases in 2012, there was a large variability in the proportion of DDD from generics across groups, with figures substantially higher for some medicines and very low proportions observed for other groups. For example, acetylsalicylic acid was the most consumed medicine for cardiovascular diseases, in line with the observed in other European countries [3], but generic medicines accounted for less than 3% of the overall number of DDD sold. On the other extreme of this spectrum is simvastatin, which was the second most consumed drug in this group, with the proportion of DDD from generics exceeding 97% in 2012. The possible reasons for the observed discrepancies deserve special attention. These may include preference of prescribers for specific pharmaceutical formulations (e.g., gastro-resistant tablets of acetylsalicylic acid or prolonged released tablets of indapamide), heterogeneity in the differences between prices of generic and non-generic medicines (e.g., there are large differences for atorvastatin or clopidogrel, whereas the prices are almost overlapping for perindopril or lisinopril) ordifferences in the availability of generic medicines across drugs or ATC groups (e.g., there is a large number of generic medicines including simvastatin, which may contribute to the large share of generics among the sales of this drug). It is also important to acknowledge a certain lack of confidence by the general public or health care professionals on the safety and efficacy of generic medicines [18, 34, 36].

Our results show that generics are responsible for only 25% of overall expenditure in this therapeutic area, reflecting their lower cost and the fact that in previous years decrease in mean cost per DDD was more pronounced for generics. Despite the fact that differences in the cost of generics and non-generics are not the same across chemical subgroups, both in absolute and relative value, the cost of non-generics also tends to decrease when generics are introduced in the market [34]. Additionally, in Portugal several measures have been adopted in recent years aiming to control National Health Service medicine expenditure. Along with increased use of generics, the reduction observed in the overall cost per DDD was also due to government decisions to reduce the price of all medicines (6% reduction in 2005, 2007 and 2010) and of generics only (30% reduction in 2008). An aggressive pricing and reimbursement legislation package was implemented, and the MoU has increased the requirements to reduce public expenditure, with repercussions throughout 2011 and 2012. This explains the relatively stable proportion of expenditure due to generics in recent years, despite the steep increase in the use of these medicines.

From 2012 onwards, all medicines started to be prescribed only by international nonproprietary name, and further increases in the proportion of generics sold could be anticipated. However, the impact of a more frequent use of generics in the overall expenditure may be smaller than the impact of reducing the use of more recent drugs and fixed-dose combinations in favor of essential medicines. However, trends towards higher proportions of essential medicines over the years have been much less pronounced than those observed for generics, and partially reflect an increase in the number of medicines classified as essential [37], and not necessarily changes in practice.

Trends in the sales of statins and agents acting on the renin-angiotensin system illustrate the large potential for a more rational use of medicines for prevention and treatment of cardiovascular diseases, as well as the need for formal monitoring and evaluation of the adequacy of patterns of use of medicines in accordance with clinical guidelines.

Simvastatin is the first option recommended in the Portuguese guidelines from the Directorate-General of Health, though other drugs from the same group may be used when the former is contraindicated or is ineffective, though the less expensive drugs should be selected; the 2013 edition of the “wise list” [37], also included atorvastatin in addition to simvastatin. In Portugal, these two drugs accounted for just over 50% of all DDD consumed in 2012, whereas in other European countries these drugs accounted for more than 80% or 90% of the overall number of DDD [38–42].

More than half of the top 40 most consumed medicines in Portugal are ACE inhibitors or ARB. Despite the guidelines of the Directorate-General of Health recommending preferential use of ACE inhibitors, and this is a pattern observed across several European countries [3], in Portugal the proportion of DDD from ARB is higher than from ACE inhibitors, and it has been increasing over the years. Our results also raise concern regarding frequent use of fixed-dose combinations, mainly ACE inhibitors or ARB with diuretics, whose cost per DDD is substantially higher, reaching nearly half the number of DDD of medicines from these groups, which has no parallel in other European countries [12].

In addition to its role as an instrument for promoting the efficient use of pharmacological treatments, a list of essential medicines may also provide guidance for ensuring that specific medicines in each therapeutic class remain available. For example, no medicine from the group thiazides, plain (C03AA) is currently available in Portugal, although it is part of the recommendations from the Directorate-General of Health for treatment of hypertension [43]. The reason for this unavailability deserves further attention and correction, in order not to deprive the market of valuable treatment options.

This study provides relevant information on trends in consumption of medicines for prevention and treatment of cardiovascular diseases and to define strategies aiming for more rational use at a national level, however some limitations require discussion.

The use of DDD as a unit of measurement allows direct comparison between drugs with different commercial presentations and recommended posologies, despite the DDD not necessarily reflecting the daily dose effectively prescribed, and these inaccuracies may vary along the years and across medicines; for example, the marketing authorization holder recommends a daily dose of indapamide in modified release formulations that is lower than the DDD for indapamide for oral administration. Since it is not possible to estimate, for all medicines, the extent to which the calculated number of DDD sold in Portugal is an accurate estimate of the daily doses effectively sold, to increase comparability we used the DDD recommended by the WHO, whenever available, both for trend analyses and description of the consumptions in 2012.

Our results refer only to the ambulatory use of medicines, and a more comprehensive assessment of the patterns of use of these health resources requires the use of hospital data, which represent approximately one-quarter of the overall expenditure with medicines [19]. However, the determinants of rational use at a hospital level are of a different nature, because prescription by generic name has long been used, and hospitals have a national formulary and guidelines for guiding prescriptions.

Since no list of essential medicines has been implemented in Portugal we opted to use the “wise list” for essential drug recommendations in ambulatory care issued by the *Stockholm County Pharmaceutical Committee* [29, 30] as benchmark. Although our interpretation of trends in Portugal depends upon this choice, it is unlikely that our conclusions would be different if other instrument produced under the same assumptions was adopted as reference.

We analyzed data on sales of medicines, which depend on the willingness or possibility of patients to fill their prescriptions. Although no data are available for an empirical discussion of the correlation between prescriptions and sales, a proper interpretation of some results, for example the potential to change expenditure by changing the patterns of prescription, require the assumption that there are no major differences in the relation between these measures across groups of drugs. The lack of a relation between the medicines consumed and the characteristics of the individual patients using them precludes the classification of some of the patterns identified as inappropriate use of these medicines, but the comparison with general guidelines allowed the identification of differences that can hardly be explained by specific needs of the Portuguese population.

The hypothetical scenarios of changing patterns of use of medicines defined in our study should be seen as indicative of the potential for a more rational use of these medicines, though requiring several years to be implemented, with the contribution of several stakeholders. A previous cost-saving analysis, using the mean cost per DDD in Portugal as reference, estimated potential savings of approximately 130 and 140 million € if Portugal had prescription patterns for antihypertensive and lipid-lowering drugs similar to the observed in England and Wales or Denmark, respectively [44], showing that the scenarios tested in our study are not unrealistic.

## Conclusions

In conclusion, our study shows the importance of a close monitoring of patterns of consumption of different medicines for planning strategies for a more rational use of these health resources. Specifically, we identified different targets for a potential reduction in the cost of the medicines used for prevention and treatment of cardiovascular diseases. There is margin for improvement in the use of generics, overall and for specific drugs rarely used as generics, to promote the predominant use of essential medicines, and for a more rational use of fixed-dose combinations.

## Abbreviations

€: Euro
ACE: Angiotensin-converting enzyme
ANF: National Association of Pharmacies
ARB: Angiotensin receptor blocker
ATC: Anatomic Therapeutic Chemical classification system
CEFAR: Centre for Health Evaluation & Research
CVD: Cardiovascular disease
DALYs: Disability Adjusted Life Years
DDD: Defined daily doses
MoU: Memorandum of Understanding
NA: Not applicable
STATA®: Data Analysis and Statistical Software
WHO: World Health Organization
